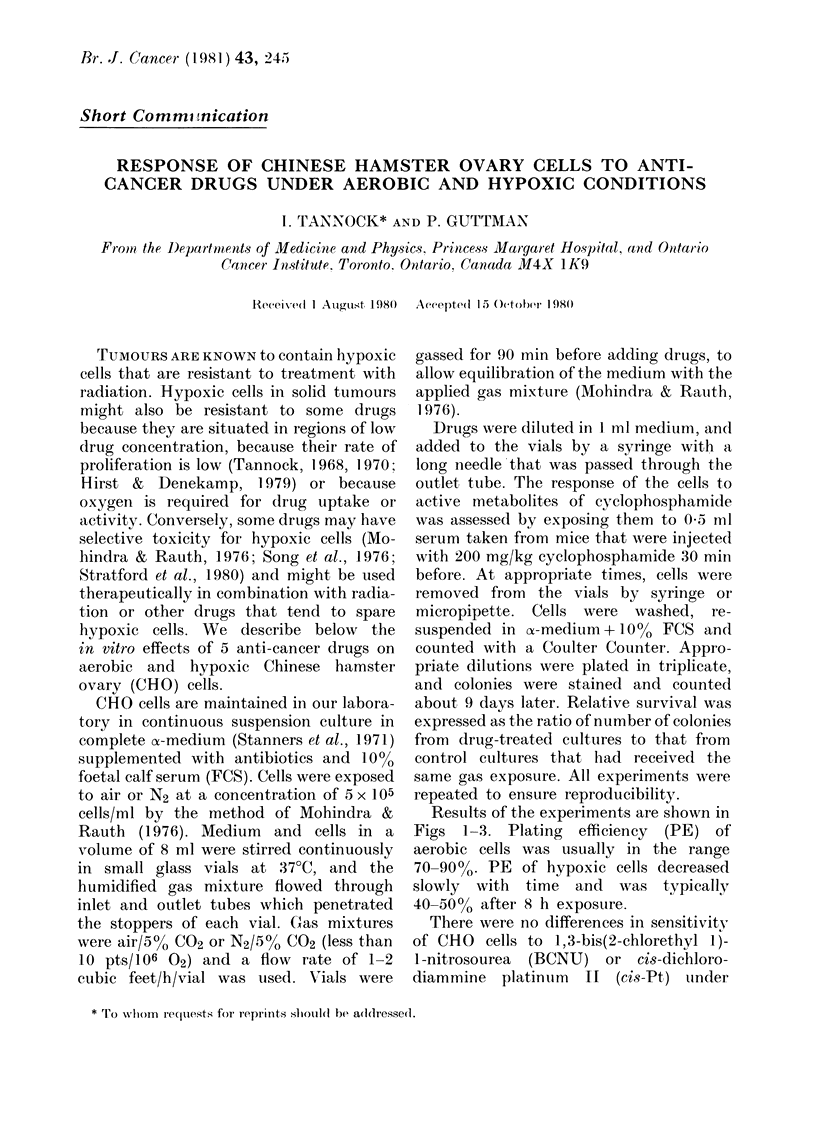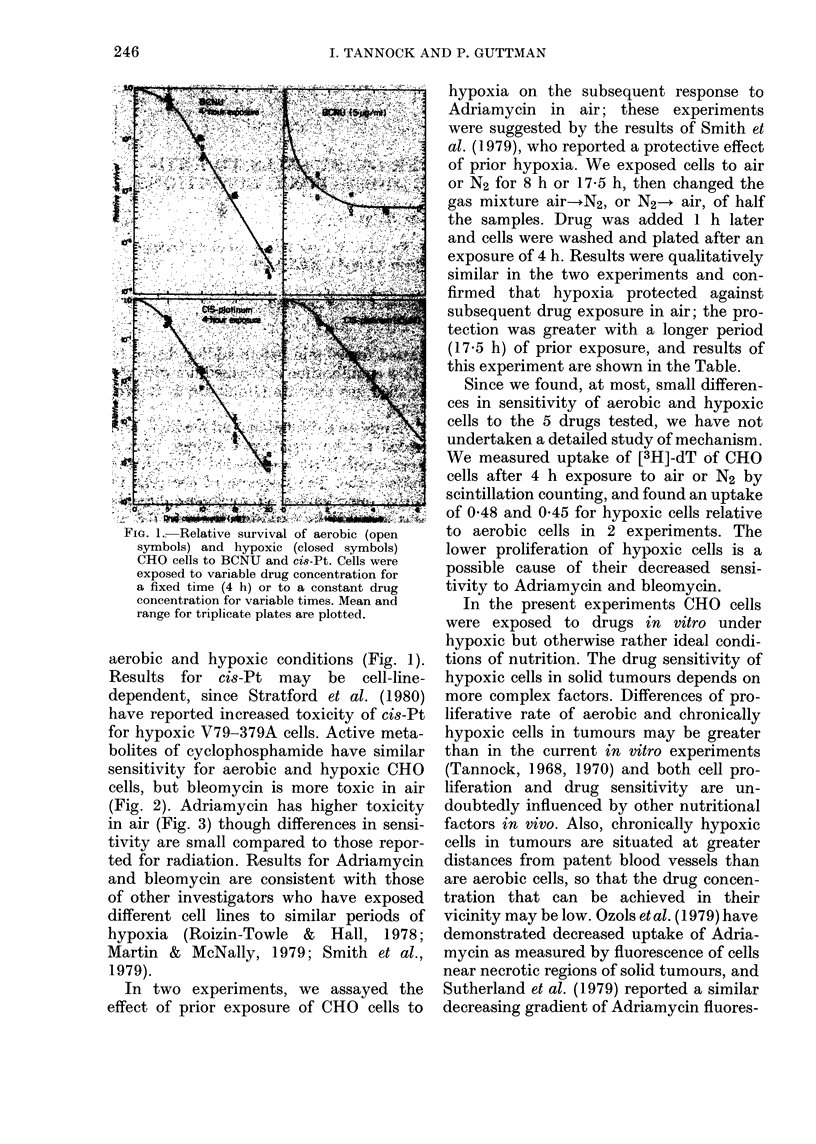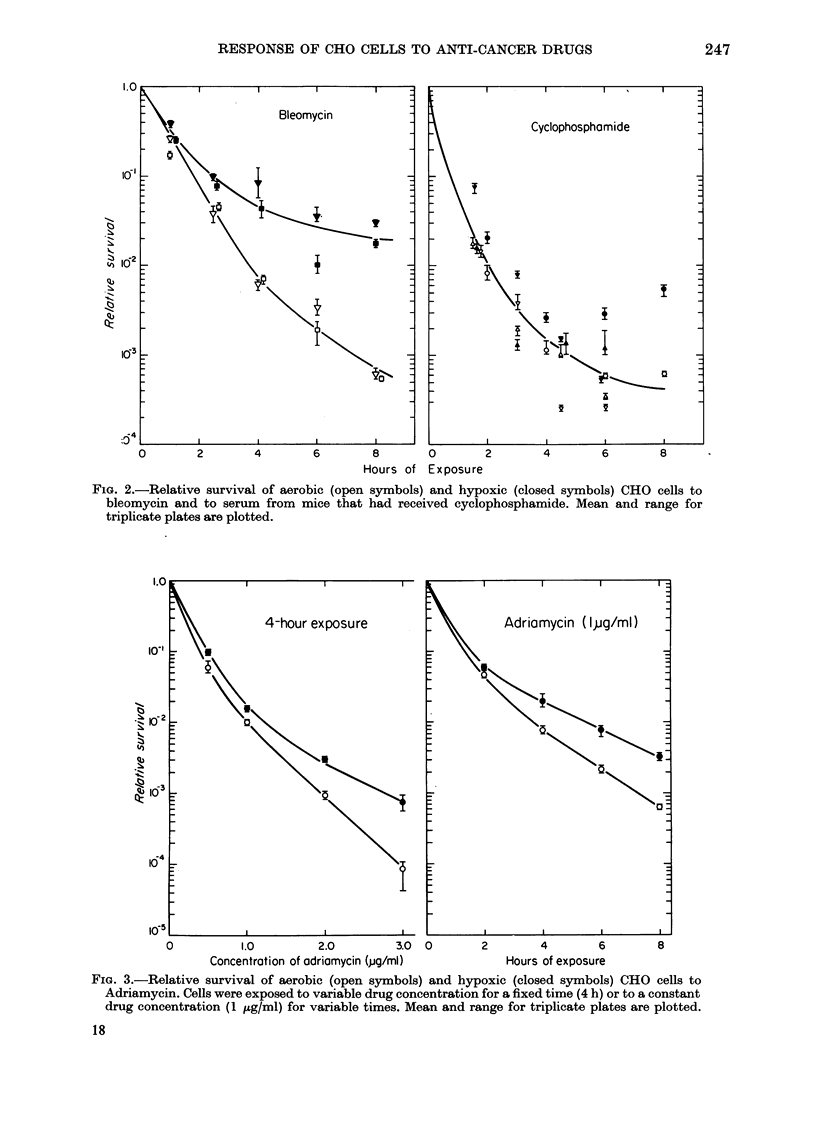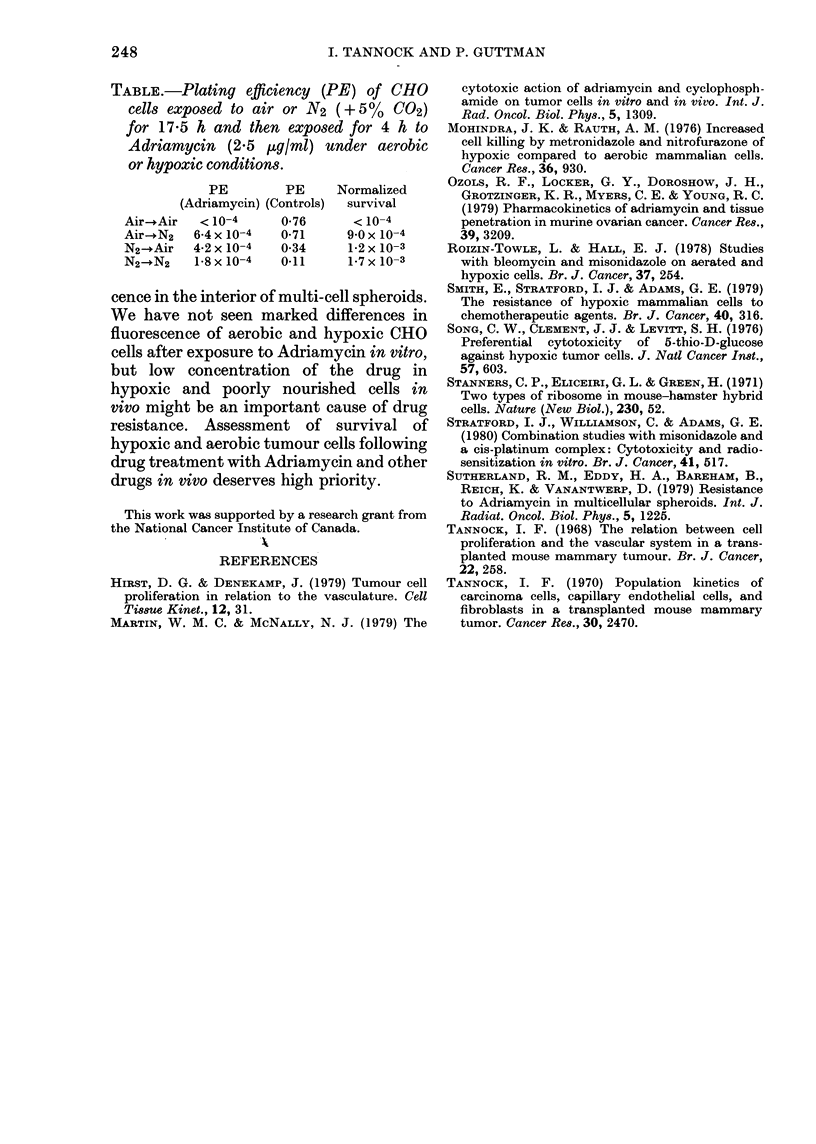# Response of Chinese hamster ovary cells to anticancer drugs under aerobic and hypoxic conditions.

**DOI:** 10.1038/bjc.1981.37

**Published:** 1981-02

**Authors:** I. Tannock, P. Guttman


*Br. J. Cancer* (1981) **43**, 245

***Short Communication***

### RESPONSE OF CHINESE HAMSTER OVARY CELLS TO ANTI-CANCER DRUGS UNDER AEROBIC AND HYPOXIC CONDITIONS

I. TANNOCK* AND P. GUTTMAN

*From the Departments of Medicine and Physics, Princess Margaret Hospital, and Ontario Cancer Institute, Toronto, Ontario, Canada M4X 1K9*

Received 1 August 1980 Accepted 15 October 1980

TUMOURS ARE KNOWN to contain hypoxic cells that are resistant to treatment with radiation. Hypoxic cells in solid tumours might also be resistant to some drugs because they are situated in regions of low drug concentration, because their rate of proliferation is low (Tannock, 1968, 1970; Hirst & Denekamp, 1979) or because oxygen is required for drug uptake or activity. Conversely, some drugs may have selective toxicity for hypoxic cells (Mohindra & Rauth, 1976; Song *et al.*, 1976; Stratford *et al.*, 1980) and might be used therapeutically in combination with radiation or other drugs that tend to spare hypoxic cells. We describe below the *in vitro* effects of 5 anti-cancer drugs on aerobic and hypoxic Chinese hamster ovary (CHO) cells.

CHO cells are maintained in our laboratory in continuous suspension culture in complete α-medium (Stanners *et al.*, 1971) supplemented with antibiotics and 10% foetal calf serum (FCS). Cells were exposed to air or $N_2$ at a concentration of $5\times10^5$ cells/ml by the method of Mohindra & Rauth (1976). Medium and cells in a volume of 8 ml were stirred continuously in small glass vials at 37°C, and the humidified gas mixture flowed through inlet and outlet tubes which penetrated the stoppers of each vial. Gas mixtures were air/5% $CO_2$ or $N_2$/5% $CO_2$ (less than 10 pts/$10^6$ $O_2$) and a flow rate of 1–2 cubic feet/h/vial was used. Vials were gassed for 90 min before adding drugs, to allow equilibration of the medium with the applied gas mixture (Mohindra & Rauth, 1976).

Drugs were diluted in 1 ml medium, and added to the vials by a syringe with a long needle that was passed through the outlet tube. The response of the cells to active metabolites of cyclophosphamide was assessed by exposing them to 0·5 ml serum taken from mice that were injected with 200 mg/kg cyclophosphamide 30 min before. At appropriate times, cells were removed from the vials by syringe or micropipette. Cells were washed, resuspended in α-medium + 10% FCS and counted with a Coulter Counter. Appropriate dilutions were plated in triplicate, and colonies were stained and counted about 9 days later. Relative survival was expressed as the ratio of number of colonies from drug-treated cultures to that from control cultures that had received the same gas exposure. All experiments were repeated to ensure reproducibility.

Results of the experiments are shown in Figs 1–3. Plating efficiency (PE) of aerobic cells was usually in the range 70–90%. PE of hypoxic cells decreased slowly with time and was typically 40–50% after 8 h exposure.

There were no differences in sensitivity of CHO cells to 1,3-bis(2-chlorethyl 1)-1-nitrosourea (BCNU) or *cis*-dichlorodiammine platinum II (*cis*-Pt) under

FIG. 1.—Relative survival of aerobic (open symbols) and hypoxic (closed symbols) CHO cells to BCNU and *cis*-Pt. Cells were exposed to variable drug concentration for a fixed time (4 h) or to a constant drug concentration for variable times. Mean and range for triplicate plates are plotted.

aerobic and hypoxic conditions (Fig. 1). Results for *cis*-Pt may be cell-line-dependent, since Stratford *et al.* (1980) have reported increased toxicity of *cis*-Pt for hypoxic V79–379A cells. Active metabolites of cyclophosphamide have similar sensitivity for aerobic and hypoxic CHO cells, but bleomycin is more toxic in air (Fig. 2). Adriamycin has higher toxicity in air (Fig. 3) though differences in sensitivity are small compared to those reported for radiation. Results for Adriamycin and bleomycin are consistent with those of other investigators who have exposed different cell lines to similar periods of hypoxia (Roizin-Towle & Hall, 1978; Martin & McNally, 1979; Smith *et al.*, 1979).

In two experiments, we assayed the effect of prior exposure of CHO cells to hypoxia on the subsequent response to Adriamycin in air; these experiments were suggested by the results of Smith *et al.* (1979), who reported a protective effect of prior hypoxia. We exposed cells to air or $N_2$ for 8 h or 17·5 h, then changed the gas mixture air→$N_2$, or $N_2$→ air, of half the samples. Drug was added 1 h later and cells were washed and plated after an exposure of 4 h. Results were qualitatively similar in the two experiments and confirmed that hypoxia protected against subsequent drug exposure in air; the protection was greater with a longer period (17·5 h) of prior exposure, and results of this experiment are shown in the Table.

Since we found, at most, small differences in sensitivity of aerobic and hypoxic cells to the 5 drugs tested, we have not undertaken a detailed study of mechanism. We measured uptake of [$^3$H]-dT of CHO cells after 4 h exposure to air or $N_2$ by scintillation counting, and found an uptake of 0·48 and 0·45 for hypoxic cells relative to aerobic cells in 2 experiments. The lower proliferation of hypoxic cells is a possible cause of their decreased sensitivity to Adriamycin and bleomycin.

In the present experiments CHO cells were exposed to drugs *in vitro* under hypoxic but otherwise rather ideal conditions of nutrition. The drug sensitivity of hypoxic cells in solid tumours depends on more complex factors. Differences of proliferative rate of aerobic and chronically hypoxic cells in tumours may be greater than in the current *in vitro* experiments (Tannock, 1968, 1970) and both cell proliferation and drug sensitivity are undoubtedly influenced by other nutritional factors *in vivo*. Also, chronically hypoxic cells in tumours are situated at greater distances from patent blood vessels than are aerobic cells, so that the drug concentration that can be achieved in their vicinity may be low. Ozols *et al.* (1979) have demonstrated decreased uptake of Adriamycin as measured by fluorescence of cells near necrotic regions of solid tumours, and Sutherland *et al.* (1979) reported a similar decreasing gradient of Adriamycin fluores-

FIG. 2.—Relative survival of aerobic (open symbols) and hypoxic (closed symbols) CHO cells to bleomycin and to serum from mice that had received cyclophosphamide. Mean and range for triplicate plates are plotted.

FIG. 3.—Relative survival of aerobic (open symbols) and hypoxic (closed symbols) CHO cells to Adriamycin. Cells were exposed to variable drug concentration for a fixed time (4 h) or to a constant drug concentration (1 µg/ml) for variable times. Mean and range for triplicate plates are plotted.

TABLE.—*Plating efficiency (PE) of CHO cells exposed to air or $N_2$ (+5% $CO_2$) for 17·5 h and then exposed for 4 h to Adriamycin (2·5 μg/ml) under aerobic or hypoxic conditions.*

| | PE (Adriamycin) | PE (Controls) | Normalized survival |
|---|---|---|---|
| Air→Air | $<10^{-4}$ | 0·76 | $<10^{-4}$ |
| Air→$N_2$ | $6·4\times10^{-4}$ | 0·71 | $9·0\times10^{-4}$ |
| $N_2$→Air | $4·2\times10^{-4}$ | 0·34 | $1·2\times10^{-3}$ |
| $N_2$→$N_2$ | $1·8\times10^{-4}$ | 0·11 | $1·7\times10^{-3}$ |

cence in the interior of multi-cell spheroids. We have not seen marked differences in fluorescence of aerobic and hypoxic CHO cells after exposure to Adriamycin *in vitro*, but low concentration of the drug in hypoxic and poorly nourished cells *in vivo* might be an important cause of drug resistance. Assessment of survival of hypoxic and aerobic tumour cells following drug treatment with Adriamycin and other drugs *in vivo* deserves high priority.

This work was supported by a research grant from the National Cancer Institute of Canada.

### REFERENCES

HIRST, D. G. & DENEKAMP, J. (1979) Tumour cell proliferation in relation to the vasculature. *Cell Tissue Kinet.*, **12**, 31.

MARTIN, W. M. C. & McNALLY, N. J. (1979) The cytotoxic action of adriamycin and cyclophosphamide on tumor cells *in vitro* and *in vivo*. *Int. J. Rad. Oncol. Biol. Phys.*, **5**, 1309.

MOHINDRA, J. K. & RAUTH, A. M. (1976) Increased cell killing by metronidazole and nitrofurazone of hypoxic compared to aerobic mammalian cells. *Cancer Res.*, **36**, 930.

OZOLS, R. F., LOCKER, G. Y., DOROSHOW, J. H., GROTZINGER, K. R., MYERS, C. E. & YOUNG, R. C. (1979) Pharmacokinetics of adriamycin and tissue penetration in murine ovarian cancer. *Cancer Res.*, **39**, 3209.

ROIZIN-TOWLE, L. & HALL, E. J. (1978) Studies with bleomycin and misonidazole on aerated and hypoxic cells. *Br. J. Cancer*, **37**, 254.

SMITH, E., STRATFORD, I. J. & ADAMS, G. E. (1979) The resistance of hypoxic mammalian cells to chemotherapeutic agents. *Br. J. Cancer*, **40**, 316.

SONG, C. W., CLEMENT, J. J. & LEVITT, S. H. (1976) Preferential cytotoxicity of 5-thio-D-glucose against hypoxic tumor cells. *J. Natl Cancer Inst.*, **57**, 603.

STANNERS, C. P., ELICEIRI, G. L. & GREEN, H. (1971) Two types of ribosome in mouse–hamster hybrid cells. *Nature (New Biol.)*, **230**, 52.

STRATFORD, I. J., WILLIAMSON, C. & ADAMS, G. E. (1980) Combination studies with misonidazole and a cis-platinum complex: Cytotoxicity and radiosensitization *in vitro*. *Br. J. Cancer*, **41**, 517.

SUTHERLAND, R. M., EDDY, H. A., BAREHAM, B., REICH, K. & VANANTWERP, D. (1979) Resistance to Adriamycin in multicellular spheroids. *Int. J. Radiat. Oncol. Biol. Phys.*, **5**, 1225.

TANNOCK, I. F. (1968) The relation between cell proliferation and the vascular system in a transplanted mouse mammary tumour. *Br. J. Cancer*, **22**, 258.

TANNOCK, I. F. (1970) Population kinetics of carcinoma cells, capillary endothelial cells, and fibroblasts in a transplanted mouse mammary tumor. *Cancer Res.*, **30**, 2470.